# Stem rust resistance in North American spring wheat to Ug99 and other virulent pathotypes of *Puccinia graminis* f. sp.* tritici*

**DOI:** 10.1007/s00122-026-05352-0

**Published:** 2026-10-27

**Authors:** Harsimardeep S. Gill, Emily Conley, Kimani Cyrus Ndung’u, Pablo Olivera Firpo, Charlotte Brault, Zennah Kosgey, Ashenafi Gemechu Degete, Sridhar Bhavani, Prabin Bajgain, Matthew N. Rouse, Yue Jin, James A. Anderson

**Affiliations:** 1 Department of Agronomy and Plant Genetics, University of Minnesota, Saint Paul, MN 55108 USA; 2 Food Crops Research Center, Kenya Agricultural and Livestock Research Organization (KALRO), Njoro, Kenya; 3 Cereal Disease Lab, United States Department of Agriculture-Agricultural Research Service, St. Paul, MN 55108 USA; 4 Debre Zeit Agricultural Research Center, Ethiopian Institute of Agricultural Research, Bishoftu, Ethiopia; 5 International Maize and Wheat Improvement Center (CIMMYT), ICRAF Campus, United Nations Avenue, P.O. Box 1041-00621 Gigiri, Nairobi Kenya; 6 U.S. Dairy Forage Research Center, United States Department of Agriculture-Agricultural Research Service, Madison, WI 53706 USA; 7 United States Department of Agriculture-Agricultural Research Service, Sugarcane Field Station, Canal Point, FL 33438 USA; 8 Department of Plant Pathology, University of Florida, Gainesville, FL 32611 USA; 9 Department of Plant Pathology, University of Minnesota, Saint Paul, MN 55108 USA

## Abstract

**Key message:**

North American spring wheat shows limited seedling resistance to a diverse set of exotic *Pgt* races but harbors valuable field resistance, including a strong locus for field resistance on chromosome 7AL.

**Abstract:**

The emergence and spread of Ug99 and other races of *Puccinia graminis* f. sp. *tritici* (*Pgt*) with virulence to widely deployed resistance genes threaten global wheat production. This study evaluated North American spring wheat germplasm for seedling responses to four *Pgt* races (TTKSK, TTKSF+, TKTTF, and TRTTF) and for field response across five environments in Ethiopia and Kenya. Seedling evaluation revealed significant race-specific variation, with 82% of the lines exhibiting resistance to race TKTTF but very limited resistance to the other races. Gene postulation based on resistance patterns suggested a high frequency of *Sr7a* (~ 92%) in this panel, with only a few lines (~ 5%) carrying *Sr8a, Sr11,* and/or *Sr31*. Field evaluations in Ethiopia and Kenya demonstrated quantitative variation for field response, with several lines showing consistent field resistance. Association mapping identified 37 significant associations for seedling resistance, representing 17 unique genomic regions. For field response, 15 significant associations were detected, including a region on chromosome arm 7AL that was consistently detected across environments. Further analysis indicated that this locus confers adult-plant resistance (APR) and might be independent of known stem rust resistance genes located in the region. Hence, KASP assay was developed to enable marker-assisted selection and further investigation of this locus. This study highlights limited seedling resistance but identifies promising sources of field resistance against exotic *Pgt* races and provides valuable insights into the genetics underlying both seedling and field responses in North American germplasm.

**Supplementary Information:**

The online version contains supplementary material available at https://doi.org/10.1007/s00122-026-05352-0.

## Introduction

Wheat stem rust, caused by the fungus *P. graminis* Pers.:Pers. f. sp. *tritici* Eriks. & E. Henn. (*Pgt*), has long been one of the most destructive diseases in the major wheat-growing regions of the world, and large regional outbreaks of stem rust can result in significant yield losses (Singh et al. 2011, 2016; Bhavani et al. 2019). Deployment of host-mediated genetic resistance is one of the most effective and sustainable ways to manage stem rust (Roelfs 1985; Singh et al. 2002, 2011). Host resistance is generally classified into all-stage resistance (ASR) and adult-plant resistance (APR). ASR is often expressed from the seedling stage and is typically conferred by race-specific, major-effect genes (*Sr* genes) that operate in a gene-for-gene manner (McIntosh et al. 1995; Singh et al. 2011; Bhavani et al. 2019). In contrast with ASR, APR is usually expressed starting around the booting stage and remains active through key developmental phases, especially during heading, flowering, and grain filling (Stuthman et al. 2007; Bajgain et al. 2016). Although APR effects are often low to moderate, APR is generally effective against a broader spectrum of *Pgt* races and is often regarded as more durable because several APR genes function in a largely race-nonspecific manner (Stuthman et al. 2007; Rauf et al. 2022). To date, more than 60 *Sr* genes have been cataloged and assigned to specific chromosomal locations, with the majority being categorized as race-specific (McIntosh et al. 1995; Karelov et al. 2022; Tong et al. 2024).

Historically, the hard red spring (HRS) wheat from North America was highly vulnerable to stem rust due to a favorable environment, large acreages of susceptible cultivars, and widespread presence of the alternate host barberry (*Berberis vulgaris*) (Roelfs 1985; Singh et al. 2002). In the Northern Great Plains, severe epidemics occurred during the early to mid-twentieth century, causing yield losses of up to 50% in Minnesota and North Dakota (Peterson 2001), with estimated economic losses reaching $356 million (Roelfs 1985). The repeated stem rust epidemics prompted intensive efforts to combat the disease, including the deployment of multiple ASR genes for host resistance and the eradication of common barberry. These widespread efforts helped significantly reduce the frequency and severity of stem rust epidemics by the 1960s, and the incidence of wheat stem rust declined for more than five decades (Stakman 1923; Roelfs 1985; Singh et al. 2002; Kolmer et al. 2007). However, wheat stem rust is now considered a re-emerging threat, as new virulent *Pgt* races have emerged, causing significant outbreaks and epidemics in Africa, Europe, and Asia over the past 25 years.

The emergence and spread of races within the Ug99 group represented the first major stem rust threat to global wheat production (Wanyera et al. 2006; Singh et al. 2008). The Ug99 race, TTKSK, first identified in Uganda in 1998, was reported to be virulent against the widely deployed gene *Sr31* (Pretorius et al. 2000; Singh et al. 2011). To date, 15 variants within the Ug99 race group have been identified, and the lineage continues to expand geographically with confirmed reports from multiple countries in East and southern Africa, Yemen, Iran, Egypt, and, more recently, Nepal in 2024 (Olivera et al. 2015, 2022a; Newcomb et al. 2016; Shahin et al. 2022; Patpour et al. 2024). In addition to the Ug99 group, several genetically distinct and highly virulent races, particularly in genetic clades III (TRTTF/TTRTF) and IV (TKTTF/TKKTF), have emerged and spread rapidly. These races have contributed to significant epidemics and outbreaks in East Africa (Olivera et al. 2015), Europe (Bhattacharya 2017; Olivera et al. 2017), the Caucasus region of Eurasia (Olivera et al. 2019), and Central Asia (Olivera et al. 2022b). These developments collectively show that pathogen populations can rapidly overcome widely deployed resistance genes and underscore the risk posed by the eventual introduction of exotic *Pgt* races into North America.

Previous screening efforts have shown that approximately 85–90% of wheat cultivars and breeding lines from North America, particularly in the HRS class, are susceptible to Ug99 and other races present in eastern Africa (Jin and Singh 2006; Rouse et al. 2011; Bajgain et al. 2015; Hiebert et al. 2017; Edae et al. 2024). Furthermore, Edae et al. (2018) reported that most modern North American spring wheat germplasm depends on combinations of race-specific *Sr* genes for resistance to contemporary North American races, and that the correlation between resistance to Ug99 and to North American races is limited. This necessitates a comprehensive characterization of resistance to exotic *Pgt* races to identify new genes and quantitative trait loci (QTL). Linkage mapping and genome-wide association studies (GWAS) have become powerful tools for dissecting the genetic architecture of complex traits, such as quantitative disease resistance in elite germplasm (Yu et al. 2006; Gupta et al. 2014). In wheat, these approaches have been used to identify loci conferring rust resistance in breeding materials, including both ASR genes and field resistance QTL, thereby accelerating their deployment (Bajgain et al. 2015, 2016; Hiebert et al. 2017; Kaur et al. 2023; Edae et al. 2024).

Building on the previous studies, the present study focused on phenotypic characterization of stem rust resistance at the seedling and field stages, along with GWAS, to identify novel sources of resistance to Ug99 and other virulent *Pgt* races using a diverse panel of breeding lines (Conley 2021; Gill et al. 2025). The specific objectives of this study were to (i) evaluate seedling and field responses of North American spring wheat to Ug99 and other virulent *Pgt* races, (ii) study race-specific resistance patterns to postulate the presence of *Sr* genes, and (iii) identify genomic regions associated with ASR and field resistance. Based on the findings, we also attempted to develop diagnostic markers to facilitate marker-assisted selection (MAS) and deployment of a locus associated with field resistance.

## Materials and methods

### Plant materials

A population of breeding lines and cultivars representing the genetic diversity of the University of Minnesota’s spring wheat breeding program was used in this study (Supplementary Table S1). The mapping panel (referred to as the MN-WGS panel) included 383 HRS wheat genotypes, comprising advanced experimental lines and 20 elite lines that are frequently used as crossing parents and appear in the pedigrees of many panel entries (Conley 2021; Gill et al. 2025). Overall, the pedigree structure of the MN-WGS panel features lines contributed by multiple spring wheat breeding programs in the North American HRS wheat region, including the University of Minnesota, North Dakota State University, South Dakota State University, and private seed companies.

### Screening for stem rust resistance

#### Seedling evaluations

The MN-WGS panel was evaluated at the seedling stage for their reaction to four races of *Pgt* with broad virulence, including TTKSK (isolate 04KEN156/04), TTKSF+ (09ZIM01-2), TKTTF (isolate 13ETH18-1), and TRTTF (isolate 06YEM34-1). As part of the USDA-ARS Cereal Disease Laboratory’s standard protocol for handling *Pgt* isolates, each isolate is evaluated annually against the stem rust differential set and an additional set of specific genes before being used for germplasm screening. This evaluation verifies the isolate’s race designation and confirms its purity. Information about the *Pgt* isolates used in this study is summarized in Table 1.

**Table 1:** Isolate designation, origin, and virulence phenotype of races of *Puccinia graminis* f. sp. *tritici* used to evaluate stem rust response at the seedling stage

Race	Isolate	Origin	Virulence/avirulence
TTKSK	04KEN156/04	Kenya	*Sr5 6 7a 7b 8a 9a 9b 9d 9e 9g 10 11 17 21 30 31 38 McN ****/**** Sr24 36 Tmp 9h*
TTKSF+	09ZIM01-2	Zimbabwe	*Sr5 6 7a 7b 8a 9a 9b 9d 9e 9g 9h 10 11 17 21 30 38 McN ****/**** Sr24 31 36 Tmp*
TKTTF	13ETH18-1	Ethiopia	*Sr5 6 7b 8a 9a 9b 9d 9e 9g 9h 10 17 21 30 36 38 McN Tmp ****/**** Sr11 24 31 7a*
TRTTF	06YEM34-1	Yemen	*Sr5 6 7a 7b 9a 9b 9d 9e 9g 10 11 17 21 30 36 38 McN Tmp**** /**** Sr9h 8a 24 31*

Race TTKSK, the first identified variant in *Pgt* clade I, known as the Ug99 race group, is virulent on the widely used genes *Sr31* and *Sr38* (Pretorius et al. 2000; Jin et al. 2007). Race TTKSF+ is one of the few variants in the Ug99 race group that is avirulent on *Sr31* but virulent on *Sr9h* (Rouse et al. 2014). TRTTF and TKTTF are representative races of major *Pgt* genetic clades (III and IV, respectively) (Olivera et al. 2015). Inoculation and disease assessments were conducted according to the procedures outlined by Rouse et al. (2011). Five seedlings per genotype were inoculated on fully expanded primary leaves 8–10 days after planting, incubated in a dew chamber at 100% humidity for 14 h in darkness, and then allowed to dry inside the chamber under fluorescent light for 4 h. Subsequently, the inoculated plants were placed in a greenhouse maintained at 21 ± 2 °C under a 16/8-h light/dark photoperiod. To detect the presence of the resistance gene *Sr15*, an additional assessment against race TTKSK was performed under a cold temperature range of 15–18 °C in a growth chamber (Gao et al. 2019) and referred to as TTKSK (CT). Infection types (ITs) were recorded 14 days post-inoculation using the 0–4 Stakman scale (Stakman et al. 1962). Infection types of 0–2 were classified as resistant; IT = 2+ to 3−, and combinations of high and low ITs were considered moderately resistant; and IT = 3 or higher were classified as susceptible. Each test was performed in a single replicate and repeated once for validation, yielding two independent replicates for each race. The presence of stem rust resistance genes was postulated on the basis of comparing low and high ITs to the already published ITs of the four *Pgt* races on the 20 differential lines and additional resistance genes at the seedling stage (Jin et al. 2008; Olivera et al. 2012, 2015).

#### Field evaluations

Field evaluation for stem rust response was conducted at two locations: (i) the international wheat stem rust nursery at the Debre Zeit Agricultural Research Center of the Ethiopian Institute of Agricultural Research (EIAR), Debre Zeit, Ethiopia, in 2016, 2017, and 2018 (hereafter Eth16, Eth17 and Eth18) and (ii) the international stem rust phenotyping platform at the Kenya Agricultural and Livestock Research Organization (KALRO), Njoro, Kenya, in 2016 and 2017 (Ken16, Ken17). At Debre Zeit, the entries were sown in two replicates of 1 m rows with 20 cm spacing between rows. To promote inoculum buildup and uniform disease pressure, spreader rows consisting of a mixture of the susceptible varieties, including “Arendeto,” “PBW343,” “Digalu,” “Local Red,” “Leeds,” and “Morocco,” were planted perpendicular to the test entries. The nursery was artificially inoculated with a bulk inoculum composed of *Pgt* races TTKSK, TKTTF, TRTTF, TTTTF, and JRCQC. At Njoro, entries were planted in two replicates of 1 m rows with 20 cm spacing. Continuous spreader rows composed of a mixture of cultivars highly susceptible to the Ug99 race (“Caccuke,” “Kenya Eagle 10,” and “Robin”), and six CIMMYT lines with the *Sr24* gene were planted to surround the test entries 1–2 weeks before the experimental plots were planted. Bulk of stem rust urediniospores from predominant Ug99 *Pgt* races that include TTKSK, TTKST, TTKTK, and TTKTT were suspended in distilled water, and approximately 1 mL of the suspension was injected into spreader plants using a hypodermic syringe. Spreaders were also planted in hill plots, perpendicular to the entries, to facilitate the buildup and spread of the disease.

Disease assessment included infection response (pustule type and size) following Roelfs (1992) and disease severity using the modified Cobb scale (Peterson et al. 1948). Infection responses were classified as resistant (R), moderately resistant (MR), moderately susceptible (MS), and susceptible (S). When multiple infection responses occurred within a genotype, all observed categories were recorded.

### Data analysis

For seedling assays, two independent replicates were performed for each race. The Stakman ITs (0–4 scale) for each replicate were converted to a linearized scale (0–9 scale) using an in-house R script reported in a previous study (Gao et al. 2016). The R script is available at https://github.com/umngao/rust_scores_conversion. An average of two replicates was used for exploratory analysis, correlation analysis, and GWAS. For field data, disease severity and infection response were used to calculate the coefficient of Infection (COI) as described by Gao et al. (2016). In each of the five field environments, the COI was calculated from disease severity and infection response, which were normalized using the average of the two rows within each environment. The COI was used for exploratory data analysis, correlation analysis, and GWAS. To obtain across-environment best linear unbiased estimates (BLUEs) for COI, a linear model was used with genotype modeled as a fixed effect and environment as a random effect. Residual errors were assumed to be independent and normally distributed. Because only a single observation per genotype was available in each environment (normalized across the two replicates), genotype-by-environment interaction effects could not be estimated and were absorbed into the residual term.

Correlation analyses for ASR and field responses were conducted separately using the cor() function in R, and the results were visualized with ggplot2 (Wickham 2016; R Core Team 2025). For seedling assays, boxplots were generated in R using the linearized Stakman scale to summarize trait distributions of resistance responses. Variation in field COI was summarized across the five field environments using a line plot generated with ggplot2 to illustrate environmental trends and highlight highly resistant lines.

### Genotyping and analysis

The MN-WGS panel was genotyped using the 90 K Illumina Infinium iSelect Array (Conley 2021). Briefly, DNA was extracted from fresh leaf tissue from a single plant at the three-leaf stage using the BioSprint 96 DNA Plant Kit following the manufacturer’s instructions. Single-nucleotide polymorphisms (SNPs) were called using the Genome Studio Polyploid Clustering Module v1.0 (Illumina, Inc., USA), following the method described by Wang et al. (2014) for unrelated lines. SNPs were subjected to quality control, with markers exhibiting more than 10% missing calls, a minor allele frequency (MAF) less than 5%, and more than 10% heterozygosity removed. The remaining set was imputed using the LD-kNNi algorithm (Money et al. 2015) implemented in TASSEL (Bradbury et al. 2007). The final dataset included 13,846 SNP markers, with genetic map positions obtained from the consensus wheat map developed from six double-haploid mapping populations (Wang et al. 2014). Physical positions for SNPs were retrieved based on the Chinese Spring wheat reference genome RefSeq v1.1 (IWGSC 2018). Population structure was previously evaluated using principal component analysis (PCA) and K-means clustering of this panel (Sallam et al. 2020), which served as a reference for performing association mapping in this study. Similarly, linkage disequilibrium (LD) in the MN-WGS panel was estimated in our previous study (Sallam et al. 2020) at the whole-genome level and for individual wheat genomes. In this study, LD decay for genomic regions of interest was analyzed in TASSEL (Bradbury et al. 2007).

### Marker–trait associations

A GWAS was conducted using 13,846 SNP markers and phenotype data from seedling assays and field evaluations. For seedling assays, association analysis was performed for each *Pgt* race using the average linear Stakman score from two independent replicates. Similarly, GWAS for field response utilized normalized COI values from each of the five independent environments. A multilocus mixed model, Fixed and random model Circulating Probability Unification (FarmCPU), was employed to test marker–trait associations (MTAs) (Liu et al. 2016). The mixed model was implemented in the Genomic Association and Prediction Integrated Tool (GAPIT) version 3.0 in R (Wang and Zhang 2021). FarmCPU has been extensively used in wheat rust studies and provides improved power and greater control over false positives compared to single-locus mixed models (Kaur et al. 2023). To account for population structure, the first three principal components (PCs) derived from the SNP dataset were included in the model. We used a Bonferroni-corrected threshold at the 5% significance (corresponding to − log_10_(*P*) = 5.4) to identify statistically significant associations. Additionally, the genomic region identified on chromosome 7AL was evaluated for its allelic effect on field resistance across environments. For each of the five environments, the two allelic groups were compared for differences in field resistance scores (COI) using a t-test. Differences between allelic groups were assessed independently for each of the five field environments, and the results were visualized using boxplots generated with the R package ggplot2.

### KASP genotyping of 1BL.1RS translocation

The MN-WGS panel was genotyped using the KASP marker *1RS:1BL_6110* (Rasheed et al. 2016) to detect the 1BL.1RS translocation and validate the presence of the rye-derived *Sr31* gene. The assay mixture was prepared with 12 µM each of the allele-specific primers and 30 µM of the common reverse primer in molecular-grade water (Sigma Cat. No. 4502). Genotyping was performed using PACE™ chemistry in 4 µL reactions containing 1.2 µL of genomic DNA (normalized to 20 ng/µL), 0.055 µL of assay mixture, 2 µL of 2X PACE™ Genotyping Master Mix (3CR Bioscience, UK), and 0.745 µL of water. Thermal cycling was conducted on a Roche LightCycler® 480 using a 384-well block with the following conditions: 95 °C for 15 min; 10 touchdown cycles of 94 °C for 20 s and 61 °C for 60 s (decreasing 0.6 °C per cycle); and 38 cycles of 94 °C for 20 s and 55 °C for 60 s. Fluorescence was read at 37 °C after a 2-min cool-down. Alleles were called using the LightCycler® 480 Endpoint Genotyping Software Module (Roche Diagnostics, Indianapolis, Indiana, USA).

### Development of KASP markers for the 7AL region

To enable use of a potential APR QTL identified on chromosome 7AL (712.1–719.6 Mbp), a KASP assay was developed to target a SNP significantly associated with the resistance phenotype across multiple environments. Allelic effect analysis identified the SNP Tdurum_contig12454_585, mapped at 719.6 Mbp on chromosome 7AL, as significant across all field environments. The probe sequences for the SNP *Tdurum_contig12454_585* were retrieved from the wheat 90 K iSelect SNP array platform (Wang et al. 2014). Allele-specific forward primers and common reverse primers were designed based on these probe sequences using the PACE® assay design service provided by 3CR Bioscience (United Kingdom). Genotyping was performed using the PACE™ chemistry and thermal cycling conditions described above. To validate the specificity of the newly developed assay, genotype calls from the KASP marker were compared with the original iSelect 90 K SNP array data for overlapping panel lines. The primer sequences and genomic coordinates for these assays are listed in Supplementary Table S2.

## Results

### Seedling resistance to stem rust

**Fig. 1:**
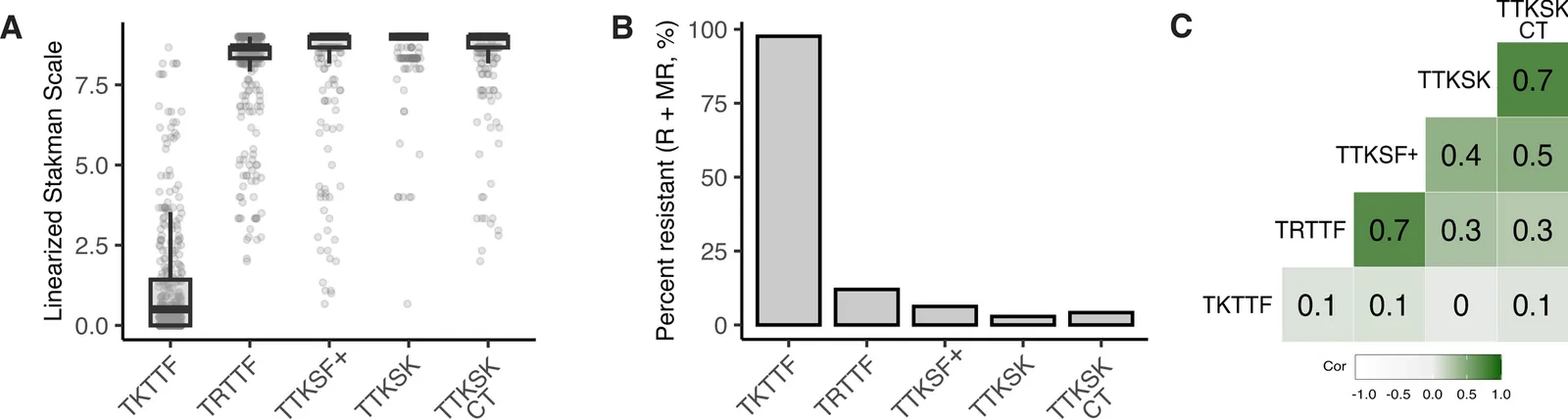
Seedling responses of the MN-WGS panel to *Puccinia graminis* f. sp. *tritici* (*Pgt*) races. **A** Boxplots presenting the distribution of seedling infection types to races TKTTF, TRTTF, TTKSF+, TTKSK, and TTKSK (CT; cold temperature assay) plotted as linearized Stakman scale (0–9). **B** Percentage of lines identified as resistant or moderately resistant (R + MR) for each race (including TTKSK-CT). **C** Pairwise correlations among race responses based on linearized Stakman scores

Seedling IT assays revealed differences in virulence among the evaluated *Pgt* races, with TKTTF being the least virulent and TTKSK the most virulent (Fig. 1A). We used the Stakman scale to classify the 383 lines of the MN-WGS panel into resistant, moderately resistant, and susceptible classes (Fig. 1B; Table 2). Among the four races, TKTTF was the least virulent, with 314 lines (82%) showing resistant ITs, 60 lines (15%) showing moderately resistant ITs, and only nine lines (2%) showing susceptible reactions. In contrast, TRTTF and TTKSF+ mostly produced susceptible responses, with 337 (~ 88%) and 358 (~ 94%) lines being susceptible, respectively. Race TTKSK exhibited the highest virulence, and most of the lines screened for ASR against TTKSK were susceptible. For instance, 368 lines (~ 97%) exhibited a susceptible response to TTKSK, whereas only 11 lines showed resistant or moderately resistant responses. Additionally, TTKSK exhibited a similar virulence pattern under cold treatment, with 366 lines (95%) rated as susceptible and only 16 as resistant or moderately resistant (Fig. 1B and Table 2). Among the 383 lines, five showed a highly resistant response to all four races (Supplementary Table S3). Furthermore, we identified 17 lines that were susceptible to TTKSK but showed resistance to TKTTF, TRTTF, and TTKSF+ (Supplementary Table S3).

**Table 2:** Summary of seedling infection types to *Pgt* races (TKTTF, TRTTF, TTKSF+, and TTKSK), including the cold temperature TTKSK assay

Race	Total	Resistant (R)	Moderately resistant (MR)	Susceptible
TKTTF	383	314	60	9
TRTTF	383	30	16	337
TTKSF+	382	19	5	358
TTKSK	379	6	5	368
TTKSK (CT)	382	10	6	366

The pairwise correlations among the races ranged from low to moderate (Fig. 1C). The responses of the lines to race TTKSK were moderately correlated with those to races TRTTF (*r* = 0.3; *P* < *0.001*) and TTKSF+ (*r* = 0.4; *P* < *0.001*). Additionally, responses to race TTKSF+ were significantly correlated with responses to TRTTF (*r* = 0.7; *P* < *0.001*). As expected, a strong correlation was observed between responses to the two TTKSK treatments (*r* = 0.7; *P* < *0.001*). In contrast, responses to race TKTTF showed a weak correlation with responses to all other races (Fig. 1C).

### Gene postulation based on seedling evaluation

To further understand the genetics underlying *Pgt* resistance in our panel, we used the expected race-specific resistance patterns and ITs from seedling assays for four races to postulate the known race-specific genes in the MN-WGS panel (Table 3 and Supplementary Table S3). The race-specific pattern showed that *Sr7a* (Knott and Anderson 1956) was the most common ASR gene in the MN-WGS panel, with gene postulation indicating its presence in 354 of 383 lines. Additionally, gene postulation indicated the presence of a few other *Sr* genes at a low frequency, with or without *Sr7a* (Supplementary Table S3), including *Sr8a* (Knott and Anderson 1956) in 20 lines, *Sr11* (Knott and Anderson 1956; Nirmala et al. 2016) in 20 lines, and *Sr31* (McIntosh et al. 1995) in 15 lines. *Sr31* was postulated mostly in combination with *Sr7a* and/or *Sr11*. We also genotyped the MN-WGS panel with a KASP marker for 1BL.1RS translocation (Supplementary Table S3). Of the 15 lines postulated to carry *Sr31*, 14 were positive for the 1BL.1RS translocation. Additionally, we postulated other genes, including *Sr15* (Watson and Luig 1966) and *Sr1RS*^*Amigo*^, at very low frequencies, with only two lines postulated to carry them.

**Table 3:** Postulated stem rust resistance genes based on seedling infection type patterns across four *Pgt* races (TKTTF, TRTTF, TTKSF+, and TTKSK). N denotes the approximate number of lines believed to carry each respective gene

Postulated *Sr* gene	Effectiveness	Chromosome	N
*Sr7a*	TKTTF	4AL	354
*Sr8a*	TRTTF	6AS	20
*Sr11*	TKTTF	6BL	20
*Sr31*	TKTTF, TRTTF, TTKSF+	1BL.1RS	15
*Sr1RS*^*Amigo*^	TKTTF, TTKSK, TTKSF+	1AL.1RS	< 5

### Field response to stem rust in African nurseries

**Fig. 2:**
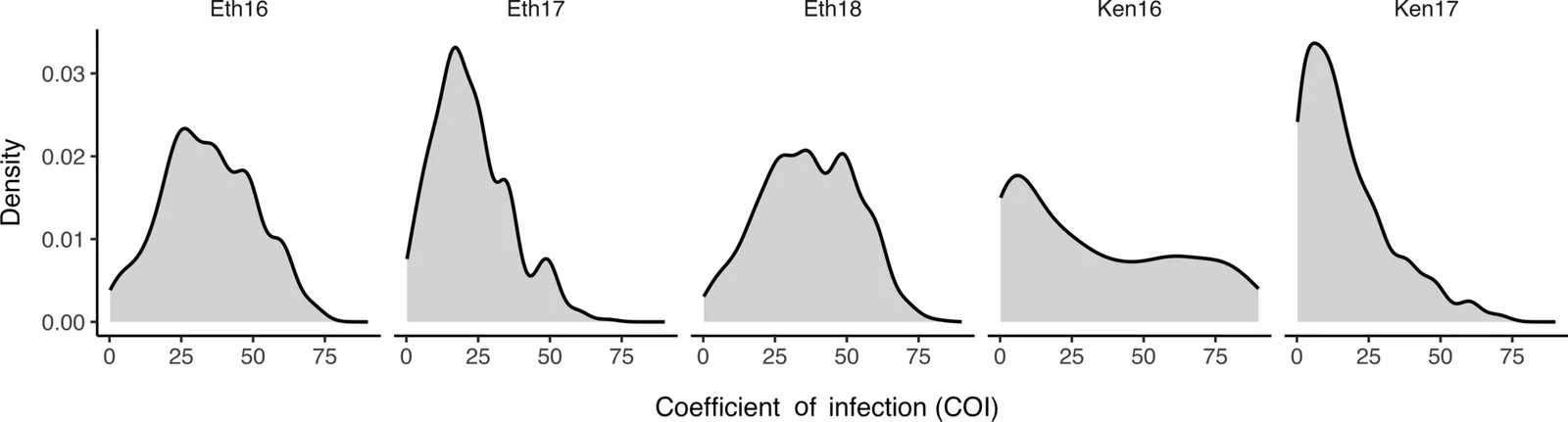
Distribution of field response to stem rust across five field environments in Ethiopia (Eth16, Eth17, and Eth18) and Kenya (Ken16 and Ken17)

**Fig. 3:**
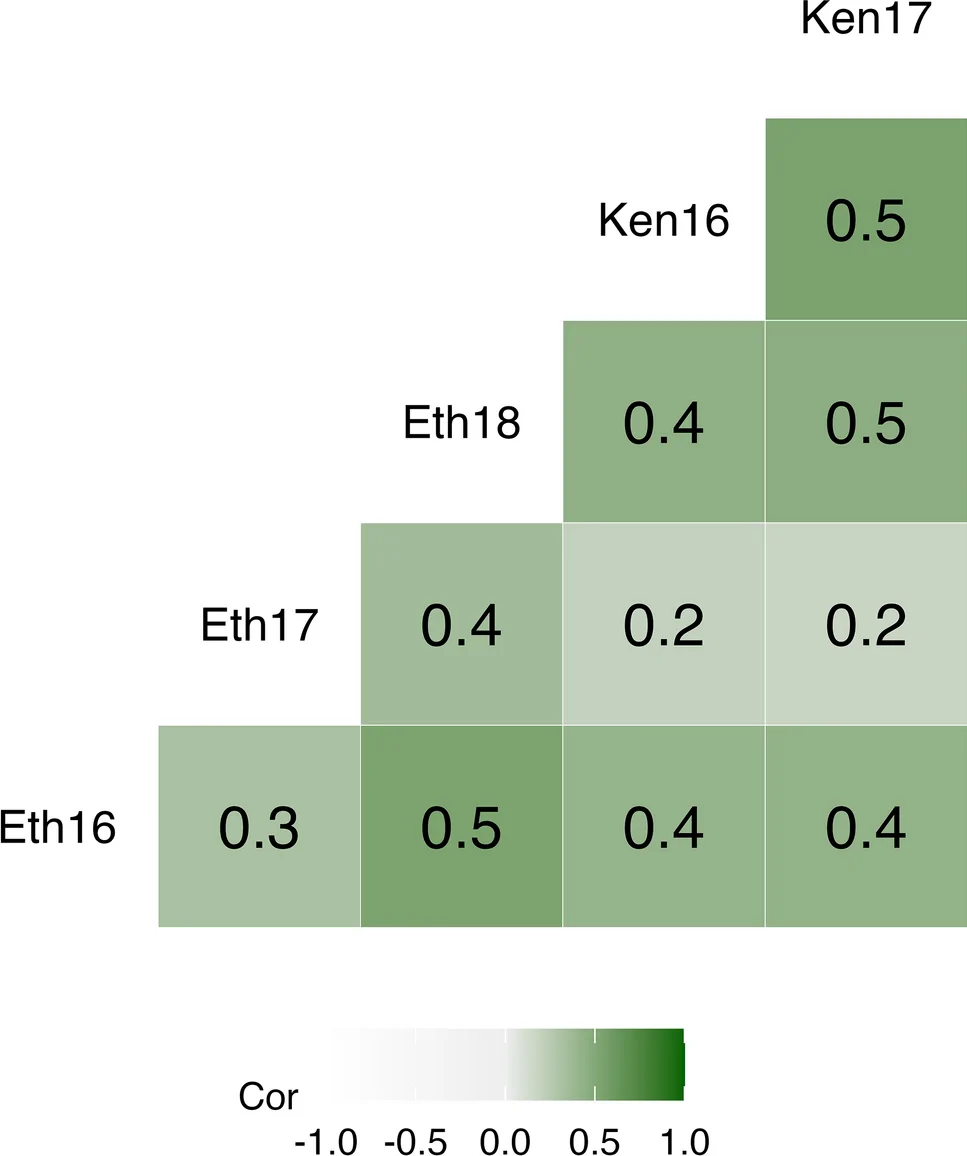
Pairwise correlations among field resistance responses to stem rust across five field environments in Ethiopia (Eth16, Eth17, and Eth18) and Kenya (Ken16 and Ken17)

**Fig. 4:**
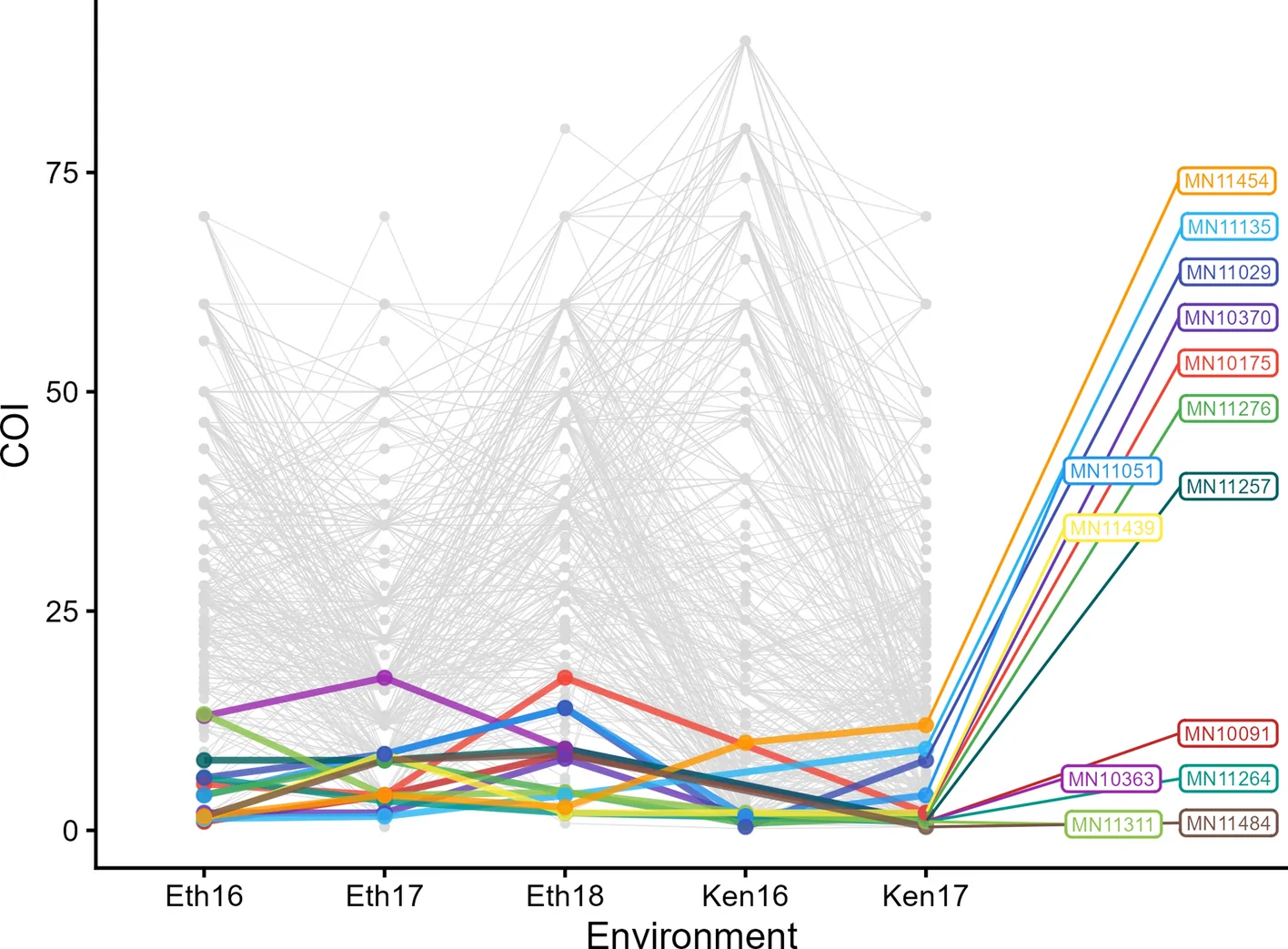
Stability of field resistance across five field environments. Coefficient of infection (COI) values for individual lines evaluated in Ethiopia (Eth16, Eth17, and Eth18) and Kenya (Ken16 and Ken17) are shown as connected line plots across environments (in gray). Colored lines and points highlight lines with consistently low COI across environments

The distribution of field response showed significant variation across the five environments, with mean COI values ranging from 17.2 to 36.6 (Fig. 2 and Table 4). The lowest disease severity was observed in Ken17, with a mean COI of 17.2, while Eth18 had the highest severity with a mean COI of 36.6 (Table 4). Overall, the Kenyan environments exhibited greater variation in field response, with Ken16 showing a wider distribution of disease scores, whereas Ken17 displayed a narrower distribution dominated by low COI values. In comparison, the three Ethiopian nurseries had more consistent disease distributions, with standard deviations ranging from 13.2 to 16.4. Moderate correlations were observed among the field environments (Fig. 3). Pairwise correlations among environments ranged from *r* = 0.2 to *r* = 0.5, with significant correlations observed between Eth16 and Eth18 (*P* < *0.05*) and between Ken16 and Ken17 (*P* < *0.05*), indicating that genotypic performance for field response was moderately stable across years and locations. Eth17 showed a comparatively weaker association with the other four environments (Fig. 3). Furthermore, we identified lines exhibiting stable field resistance across five field environments in Kenya and Ethiopia, with at least 14 lines showing consistent low COI values (in the bottom 10%) across multiple environments (Fig. 4). Disease severities across individual environments and pedigree information for these lines are provided in Supplementary Tables S1 and S4.

**Table 4:** Descriptive statistics (mean, standard deviation, minimum, and maximum) for field response (expressed as coefficient of infection) for the MN-WGS panel evaluated in Eth16, Eth17, Eth18, Ken16, and Ken17

Environment	*N*	Mean	SD	Min	Max
Eth16	376	34.4	16.0	1.0	70
Eth17	381	22.4	13.2	0.4	70
Eth18	381	36.6	16.4	0.8	80
Ken16	331	33.5	28.7	0.2	90
Ken17	379	17.2	15.2	0.4	70

### Genetic mapping of seedling resistance

**Fig. 5:**
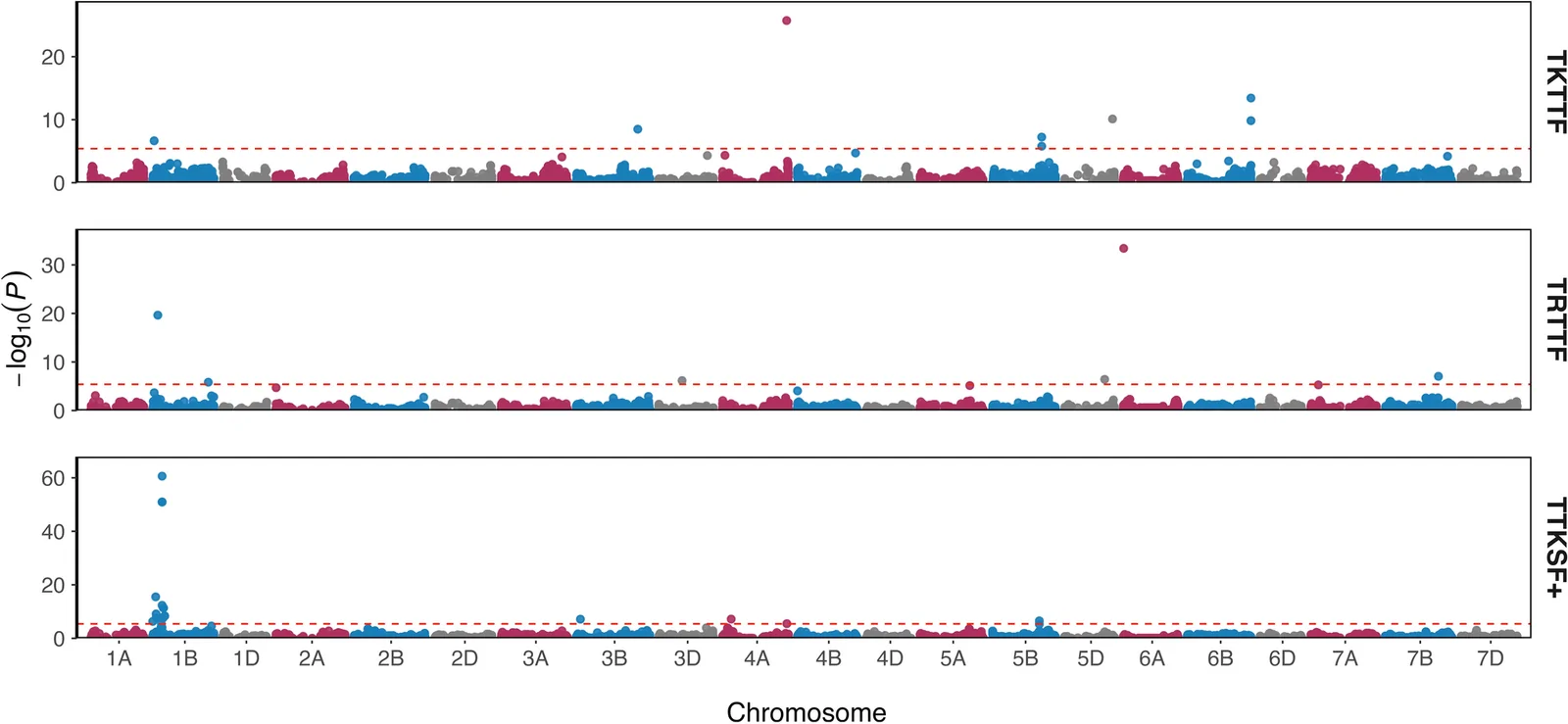
Manhattan plots presenting the genome-wide association results for seedling resistance to *Pgt* races TKTTF, TRTTF, and TTKSF+. The horizontal line indicates the Bonferroni-corrected significance threshold, and markers exceeding this threshold were considered significant marker–trait associations

Across all races, 37 MTAs surpassing the Bonferroni-corrected threshold (− log_10_(*P*) = 5.4) were identified, representing 17 unique genomic regions distributed across chromosomes 1B, 3B, 3D, 4A, 5B, 5D, 6A, 6B, and 7B (Fig. 5 and Supplementary Table S5). The number of distinct genomic regions associated with resistance was six for TKTTF and TRTTF, and five for TTKSF+. We did not identify any MTA for TTKSK at the Bonferroni-corrected threshold.

A total of eight MTAs, corresponding to six distinct genomic regions, were identified on chromosomes 1B, 3B, 4A, 5B, 5D, and 6B for race TKTTF. The most significant marker *Excalibur_c13811_1086*; (− log_10_(*P*) = 25.7) was identified on the long arm of chromosome 4A. A strong resistance region for TKTTF was also located on chromosome 6BL, encompassing SNPs *BobWhite_c7810_102* and *BobWhite_c13202_312*.

For race TRTTF, six MTAs corresponding to six distinct genomic regions were detected on chromosomes 1B, 3D, 5D, 6A, and 7B. The most significant association for TRTTF was detected on the short arm of chromosome 6A (*Tdurum_contig12045_1230*; − log_10_(*P*) = 33.4), followed by another association on chromosome 1BS (*CAP8_rep_c9642_82*; − log_10_(*P*) = 19.6). The highest number of MTAs (*n* = 23) was identified for race TTKSF+, with 19 of these 23 MTAs localized to the short arm of chromosome 1B. Among these 19 MTAs on 1BS, a SNP at approximately 106 Mbp (*BS00001364_51*) was the most significant (− log_10_(*P*) = 60.6). Additionally, four other MTAs for TTKSF+ were detected on chromosomes 3B, 4A, and 5B (Supplementary Table S5).

### Genetic mapping of field resistance

**Fig. 6:**
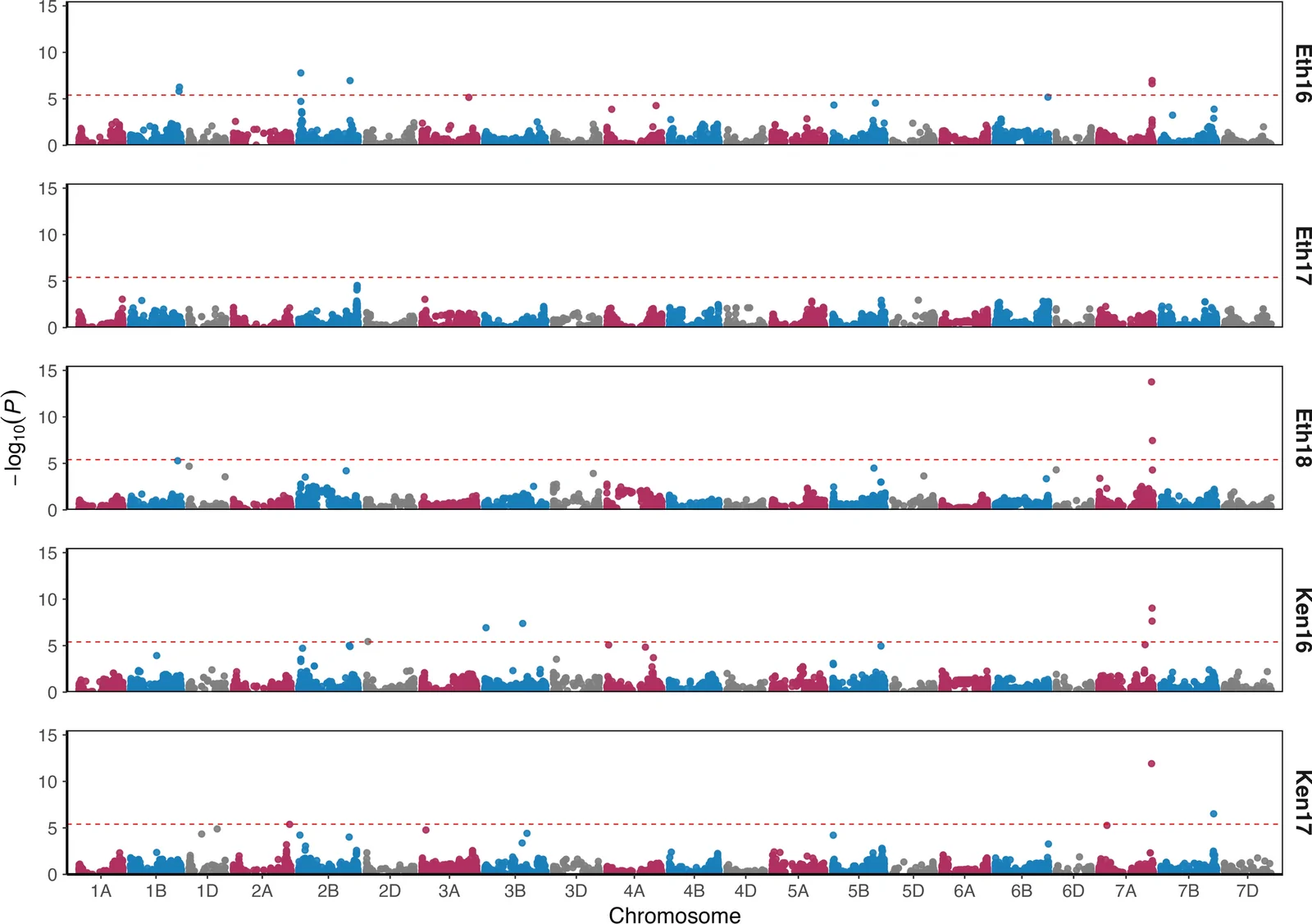
Manhattan plots showing marker–trait associations for field resistance evaluated in five environments (Eth16, Eth17, Eth18, Ken16, and Ken17). The horizontal line indicates the Bonferroni-corrected significance threshold, and markers exceeding this threshold were considered significant marker–trait associations

A total of 15 significant MTAs (− log_10_(*P*) > 5.4) were identified on chromosomes 1B, 2B, 2D, 3B, 7A, and 7B, including multiple environment-specific associations and one major region (chromosome 7A) consistently detected across environments (Table 5 and Fig. 6). The number of significant MTAs was six, two, five, and two in Eth16, Eth18, Ken16, and Ken17, respectively, with no associations identified in Eth17 (Table 5).

**Table 5:** Significant marker–trait associations for field resistance across five environments, including SNP identifier, chromosome (Chr) and physical position (Pos; based on IWGSC RefSeq 1.1), minor allele frequency (MAF), and the negative log_10_ of the *P* value for each association

Env	SNP	Chr	Pos	MAF	−log_10_(*P*)
Eth16	*BS00028476_51*	1B	654,809,199	0.10	5.8
Eth16	*BS00015519_51*	1B	660,528,071	0.12	6.3
Eth16	*Excalibur_c27388_252*	2B	29,991,110	0.38	7.8
Eth16	*wsnp_RFL_Contig2123_1397739*	2B	692,468,817	0.15	7.0
Eth16	*Tdurum_contig12454_585*	7A	719,562,931	0.44	6.6
Eth16	*Kukri_c9728_1171*	7A	719,567,332	0.28	7.0
Eth18	*Ku_c19745_892*	7A	712,058,659	0.45	13.8
Eth18	*wsnp_CAP11_c298_250917*	7A	724,086,064	0.44	7.5
Ken16	*Kukri_c27309_590*	2D	31,806,310	0.24	5.4
Ken16	*IAAV3924*	3B	20,450,724	0.49	6.9
Ken16	*BS00108055_51*	3B	513,987,620	0.15	7.4
Ken16	*Tdurum_contig12454_585*	7A	719,562,931	0.40	9.0
Ken16	*BS00010677_51*	7A	719,577,009	0.40	7.6
Ken17	*Ku_c19745_892*	7A	712,058,659	0.46	11.9
Ken17	*Tdurum_contig8402_638*	7B	711,165,419	0.34	6.5

A detailed analysis showed that two of the six associations identified in Eth16 (*BS00015519_51* on 1BL and *Excalibur_c27388_252* on 2BS) also appeared in other environments when a lower significance threshold was applied (Fig. 6). Additionally, seven MTAs on the long arm of chromosome 7A were highly significant across four of the five field environments, with – log_10_(*P*) values reaching up to 13.8 (Table 5). These MTAs were represented by five distinct SNPs (*Ku_c19745_892*, *Tdurum_contig12454_585*, *Kukri_c9728_1171*, *BS00010677_51*, and *wsnp_CAP11_c298_250917*) located between approximately 712 and 724 Mbp. Pairwise LD analysis among these SNPs indicated that multiple MTAs likely correspond to a regional signal within the 712.1–719.6 Mbp interval. In particular, *Ku_c19745_892* at 712.1 Mbp showed moderate-to-strong LD with markers near 719.6 Mbp, including *Tdurum_contig12454_585* and *BS00010677_51*, while the closely positioned markers *Tdurum_contig12454_585* and *BS00010677_51* showed very high LD (Supplementary Table S6). However, the SNP *wsnp_CAP11_c298_250917* at ~ 724.1 Mbp showed weaker LD with the upstream markers, indicating that this association may represent either a distal component of the same region or a partially independent signal.

**Fig. 7:**
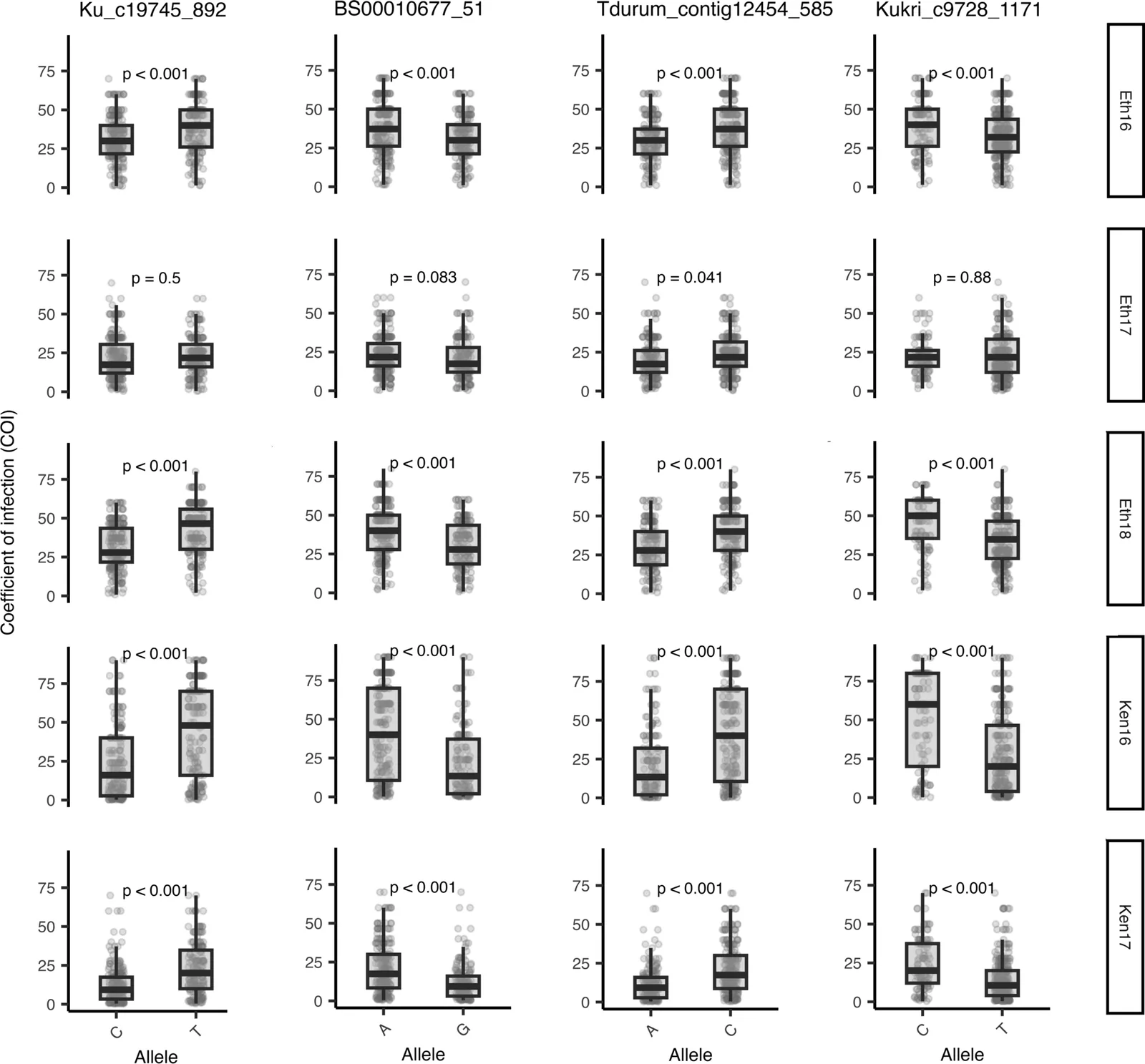
Allelic effects of significant SNPs representing the 7AL locus across five field environments. Boxplots show the distribution of the coefficient of infection for lines carrying each allele at four representative SNPs. The panels are arranged by SNPs in columns and by environment in rows (Eth16, Eth17, Eth18, Ken16, and Ken17). *P-values* above each panel indicate the significance of COI differences between allelic groups within each environment

To validate the allele-specific effects of the four SNPs spanning the 712.1–719.6 Mbp interval, we compared COI scores between the two allele classes for each SNP across the five field environments using pairwise t-tests. The four SNPs within the primary 712.1–719.6 Mbp interval showed significant allelic differentiation in most environments (Fig. 7). *Ku_c19745_892*, *BS00010677_51*, and *Kukri_c9728_1171* were significant in Eth16, Eth18, Ken16, and Ken17, but not in Eth17, whereas *Tdurum_contig12454_585* showed significant allelic effects across all five environments, supporting a robust allelic effect for the 712.1–719.6 Mbp interval (Fig. 7).

### KASP marker for 7AL locus

Based on t-tests, we identified that the SNP *Tdurum_contig12454_585*, mapped at 719.6 Mbp on chromosome 7AL, showed significant allelic differences across all field environments, with a marginally significant difference in Eth17 (Fig. 7). Therefore, this SNP was chosen to develop a KASP assay for marker-assisted selection of the 7AL locus. The developed KASP marker displayed a distinct clustering that reliably differentiated the two allelic groups (graph included in Supplementary Table S2). Technical validation of the specificity of the KASP marker using the MN-WGS panel showed high concordance (99%) between KASP genotypes and 90 K array SNP calls, confirming the robustness of this marker for fine-mapping and marker-assisted selection.

## Discussion

Screening of the MN-WGS panel for ASR revealed a low frequency of resistance to Ug99 and other virulent foreign races, except for TKTTF. According to Edae et al. (2018), most North American spring wheat breeding programs have deployed combinations of race-specific *Sr* genes that confer resistance to North American races, including QFCSC, QTHJC, TPMKC, and RCRSC. However, the correlation between resistance to Ug99 and to North American races is limited, rendering most North American spring wheat germplasm vulnerable to the exotic races. Although we identified fewer genotypes resistant to TTKSK, TTKSF+, and TRTTF, the findings provide valuable information on the presence of resistant genes in the adapted backgrounds that are effective against these exotic races and can serve as sources in the varietal development pipeline.

The overall pattern of resistance is consistent with that reported for North American spring wheat in the previous studies (Jin and Singh 2006; Rouse et al. 2011; Bajgain et al. 2015; Hiebert et al. 2017; Edae et al. 2024). Preliminary evaluations of Canadian spring wheat suggest more frequent resistance to TRTTF and limited resistance against Ug99 (Hiebert et al. 2017). Similarly, Bajgain et al. (2015) screened a relatively diverse spring wheat germplasm from North America and reported a higher frequency of resistance to TKTTF (63%) and TRTTF (44%) than to TTKSK (6%). Edae and Rouse (2020) also screened a more diverse set of North American spring wheat germplasm and reported a low frequency of resistance to TTKSK and a moderate frequency of resistance to TRTTF. In this study, we found that approximately 97%, 12%, 6%, and 3% of our germplasm were resistant or moderately resistant to TKTTF, TRTTF, TTKSF+, and TTKSK, respectively.

Gene postulation based on race-specific patterns indicated that *Sr7a* was present in 354 of 383 lines. This explains the high frequency of lines resistant to race TKTTF in seedling evaluations, as this race is avirulent on *Sr7a* (Olivera et al. 2015). *Sr11* is also effective against TKTTF, which explains the resistance response to this race in lines lacking *Sr7a*. Conversely, spring wheat breeding programs in Canada and the Pacific Northwest may carry *Sr8a* (McIntosh 1972) at higher frequencies, which provides resistance to race TRTTF. On the other hand, *Sr8a* was present at low frequency in our panel, and its frequency correlates with lines that showed resistance to the TRTTF race. Although ineffective for TTKSK, *Sr31* remains effective for TKTTF, TRTTF, and TTKSF+ (Bajgain et al. 2015; Olivera et al. 2015) and was postulated in 15 lines, mostly in combination with *Sr7a* and/or *Sr11*. We also identified a few lines, including MN10370, that were resistant to all four races evaluated. These lines exhibited low infection types (2- and 2), which are characteristics of resistance genes *Sr22* (Kerber and Dyck 1973) and *Sr24* (McIntosh et al. 1977). Because both genes are effective against all four races (Jin et al. 2007; Olivera et al. 2015), it is possible that one or both are present in these resistant lines. However, postulation of both genes was not possible based solely on the pathogen races used in this study. Therefore, molecular marker analysis is required to confirm the presence of *Sr22* and/or *Sr24* in these resistant lines.

We observed significant variation in field response, with multiple lines exhibiting resistance across five environments in Ethiopia and Kenya. Our results align with previous reports in which several elite spring wheat lines exhibited field resistance across African environments, highlighting the presence of small- to moderate-effect field resistance loci in adapted germplasm. A survey of North American spring wheat germplasm by Rouse et al. (2011) showed that several lines exhibiting high seedling ITs to TTKSK nonetheless remained resistant in the field. Similar findings were reported by Bajgain et al. (2015), where they observed significant quantitative variation for APR across African environments despite the low frequency of resistance to TTKSK in seedling evaluations. We also identified very few lines showing seedling resistance to the races TRTTF (~ 12%), TTKSF+ (~ 6%), and, particularly, TTKSK (~ 3%). However, the number of lines showing field resistance in African field environments was promising, as it has been suggested that genotypes displaying seedling susceptibility but expressing resistance at the adult-plant stage may carry loci associated with durable forms of resistance (Singh et al. 2008). Rouse et al. (2011) observed that the majority of lines identified as resistant in African field nurseries were also resistant to North American isolates in field environments and suggested that these lines may carry APR loci effective against a broad range of isolates.

GWAS results for seedling resistance also confirmed the presence of race-specific genes effective against TKTTF, TRTTF, and TTKSF+. For TKTTF, the two strongest associations identified on chromosomes 4AL and 6BL correspond to the locations of *Sr7a* and *Sr11*, respectively. Both the 4AL and 6BL regions were also identified as significant associations for resistance to TKTTF in a previous study using a more diverse set of North American germplasm (Edae and Rouse 2020). Additionally, we also detected an MTA for TKTTF on chromosome 1BS, which harbors *Sr31*. The strongest MTA for TRTTF was identified in the *Sr8a* region on chromosome 6AS, whereas the second strongest signal corresponded to the location of *Sr31* on chromosome 1BS. Furthermore, we identified two MTAs for TRTTF on chromosomes 1B and 5D, which are likely similar to those reported by Bajgain et al. (2015) for resistance to the same race. In addition, three MTAs for TRTTF on chromosomes 1B and 6A were mapped in the vicinity of previously identified MTAs for resistance to race TTRTF, which is closely related to TRTTF (Edae and Rouse 2020; Negash et al. 2022). This suggests that the same loci might confer resistance against different but closely related *Pgt* races.

For TTKSF+, we detected multiple MTAs on chromosome 1BS; however, these associations are likely specific to *Sr31* only. *Sr31* originated from *Secale cereale* cv Petkus through the 1BL.1RS translocation (Mettin et al. 1973). Due to limited recombination in this region, these signals likely represent the same 1RS haplotype in strong linkage disequilibrium, and the multiple MTAs on 1BS are probably tagging *Sr31* rather than independent resistance loci. We did not find any significant MTA for TTKSK, likely due to the limited number of resistant lines. A few lines showed low infection types (2- and 2) to multiple races, including TTKSK, which are characteristics of resistance genes *Sr22* and *Sr24*; however, their frequencies were too low to be detected by GWAS. Overall, the mapping of seedling resistance indicates that North American germplasm harbors few known *Sr* genes and some potentially uncharacterized loci that may contribute to race-specific resistance to exotic *Pgt* races. Furthermore, the strong MTAs corresponding to *Sr7a*, *Sr8a*, and *Sr11* could be useful for developing KASP markers to select these genes.

Across all field environments, we identified 15 significant MTAs for field-based response on chromosomes 1B, 2B, 2D, 3B, 7A, and 7B, representing eight unique genomic regions and indicating the presence of multiple small- to moderate-effect resistance loci in the MN-WGS panel. The lower number of associations may be due to the stringent Bonferroni threshold used in our study, which could have resulted in some false negatives. In a previous study, Bajgain et al. (2015) also reported MTAs for field response in African nurseries on chromosomes 1B, 2B, 2D, 3B, and 7B. Furthermore, the MTA on 1BL, detected in multiple environments at lower significance (Fig. 6), was also identified by Rauf et al. (2022) and is located near the *Sr58* gene.

GWAS for field response identified a stable genomic region on chromosome 7AL (spanning from 712.1 to 719.6 Mbp) that was highly significant in four of the five field environments in Ethiopia and Kenya. Three previous studies have mapped a locus in the same region associated with field response in African nurseries (Babiker et al. 2015; Pujol et al. 2015; Bokore et al. 2021). Additionally, *Sr15* (Watson and Luig 1966) and *Sr22* (Olson et al. 2010) are two ASR genes located near the 7AL association. Importantly, we did not detect any association within the same genomic interval for seedling responses to TTKSK or other *Pgt* races, indicating that the 7AL locus is unlikely to correspond to an ASR gene. The *Sr15* gene has been reported to be ineffective against TTKSK; however, Gao et al. (2019) found that *Sr15* could be effective against TTKSK at lower temperatures. Hence, we evaluated the MN-WGS panel for seedling resistance to TTKSK at a lower temperature. Nevertheless, the results indicate that only two to three lines may carry *Sr15*, which supports the exclusion of *Sr15* as the basis of the 7AL locus. Similarly, the seedling IT pattern in a few lines appears characteristic of the resistance genes *Sr22* and *Sr24*; however, their frequencies were too low to be detected by GWAS. Two previous studies, Babiker et al. (2015) and Pujol et al. (2015), mapped a stem rust resistance locus in African field environments, which was also detected in seedling tests with TTKSK. It is likely that the 7AL locus identified in the current study could be distinct from those identified in the two previous studies. In contrast, Bokore et al. (2021) mapped a stable APR QTL (*QSr-sparc-7A*) in Kenyan environments in a biparental population (AAC-Prevail x BW961) in which both parents were susceptible to TTKSK at the seedling stage, and they concluded that the locus was unlikely to be *Sr22* or *Sr15*. As the parental lines used in that study represent the North American spring wheat class, it is likely that the *QSr-sparc-7A* and the 7AL locus identified in the current study are the same. Our results suggest that high-resolution mapping of the 7AL interval is necessary to dissect the underlying gene(s) and enable their use for durable APR.

In summary, this study comprehensively evaluated stem rust resistance at both the seedling and adult-plant stages in North American spring wheat germplasm. The results reveal limited sources of ASR against Ug99 and other African races, but promising sources of field resistance. Further, GWAS identified several loci contributing to field resistance across multiple environments, including a potential APR locus on chromosome 7AL that might be independent of major stem rust resistance genes in this genomic region. The KASP assay was developed for this locus, which showed high concordance with the genotype calls from the 90 K array. Further validation of this assay across diverse genetic backgrounds is needed to facilitate targeted introgression of this locus into wheat breeding lines to confer durable resistance.

## Supplementary Information

Below is the link to the electronic supplementary material.

Supplementary file1 (XLSX 143 KB)

## Data Availability

The phenotypic datasets generated or analyzed during the current study are included as Supplementary material.

## References

[CR1] Babiker EM, Gordon TC, Chao S et al (2015) Mapping resistance to the Ug99 race group of the stem rust pathogen in a spring wheat landrace. Theor Appl Genet 128:605–612. 10.1007/s00122-015-2456-6 25599859

[CR2] Bajgain P, Rouse M, Bulli P et al (2015) Association mapping of North American spring wheat breeding germplasm reveals loci conferring resistance to Ug99 and other African stem rust races. BMC Plant Biol 15:249. 10.1186/s12870-015-0628-9 PMC4606553 26467989

[CR3] Bajgain P, Rouse MN, Tsilo TJ et al (2016) Nested association mapping of stem rust resistance in wheat using genotyping by sequencing. PLoS ONE 11:e0155760. 10.1371/journal.pone.0155760 PMC4870046 27186883

[CR4] Bhattacharya S (2017) Deadly new wheat disease threatens Europe’s crops. Nature 542:145–146. 10.1038/nature.2017.21424 28179687

[CR5] Bhavani S, Hodson DP, Huerta-Espino J et al (2019) Progress in breeding for resistance to Ug99 and other races of the stem rust fungus in CIMMYT wheat germplasm. Front Agric Sci Eng 6:210. 10.15302/J-FASE-2019268

[CR6] Bokore FE, Cuthbert RD, Hiebert CW et al (2021) Mapping stem rust resistance loci effective in Kenya in Canadian spring wheat (*Triticum aestivum* L.) lines ‘AAC Prevail’ and ‘BW961.’ Can J Plant Pathol 43:S263–S274. 10.1080/07060661.2021.1966651

[CR7] Bradbury PJ, Zhang Z, Kroon DE et al (2007) TASSEL: software for association mapping of complex traits in diverse samples. Bioinformatics 23:2633–2635. 10.1093/bioinformatics/btm308 17586829

[CR8] Conley E (2021) Association mapping and genome-wide prediction of fusarium head blight resistance in Minnesota wheat lines. University of Minnesota

[CR9] Edae EA, Kosgey Z, Bajgain P et al (2024) The genetics of Ug99 stem rust resistance in spring wheat variety ‘Linkert‘. Front Plant Sci. 10.3389/fpls.2024.1343148 PMC10954791 38516672

[CR10] Edae EA, Pumphrey MO, Rouse MN (2018) A genome-wide association study of field and seedling response to individual stem rust pathogen races reveals combinations of race-specific genes in North American spring wheat. Front Plant Sci 9:52. 10.3389/fpls.2018.00052 PMC5797647 29441083

[CR11] Edae EA, Rouse MN (2020) Association mapping of resistance to emerging stem rust pathogen races in spring wheat using genotyping-by-sequencing. Plant Genome 13:e20050. 10.1002/tpg2.20050 PMC12806954 33217214

[CR12] Gao L, Babiker EM, Nava IC et al (2019) Temperature-sensitive wheat stem rust resistance gene *Sr15* is effective against *Puccinia graminis* f. sp. *tritici* race TTKSK. Plant Pathol 68:143–151. 10.1111/ppa.12928

[CR13] Gao L, Turner MK, Chao S et al (2016) Genome wide association study of seedling and adult plant leaf rust resistance in elite spring wheat breeding lines. PLoS ONE 11:e0148671. 10.1371/journal.pone.0148671 PMC4744023 26849364

[CR14] Gill HS, Conley E, Brault C et al (2025) Association mapping and genomic prediction for processing and end‐use quality traits in wheat (*Triticum aestivum *L.). Plant Genome 18:e20529. 10.1002/tpg2.20529 PMC11726427 39539031

[CR15] Gupta PK, Kulwal PL, Jaiswal V (2014) Association mapping in crop plants: opportunities and challenges. Advances in genetics. Academic Press Inc., pp 109–147 10.1016/B978-0-12-800271-1.00002-0 24880734

[CR16] Hiebert CW, Rouse MN, Nirmala J, Fetch T (2017) Genetic mapping of stem rust resistance to *Puccinia graminis* f. sp. *tritici* race TRTTF in the Canadian wheat cultivar Harvest. Phytopathology 107:192–197. 10.1094/PHYTO-05-16-0186-R 27705664

[CR17] IWGSC (2018) Shifting the limits in wheat research and breeding using a fully annotated reference genome. Science 361:eaar7191. 10.1126/science.aar7191 30115783

[CR18] Jin Y, Singh RP (2006) Resistance in U.S. wheat to recent Eastern African isolates of *Puccinia graminis* f. sp. *tritici* with virulence to resistance gene *Sr31*. Plant Dis 90:476–480. 10.1094/PD-90-0476 30786597

[CR19] Jin Y, Singh RP, Ward RW et al (2007) Characterization of seedling infection types and adult plant infection responses of monogenic *Sr* gene lines to race TTKS of *Puccinia graminis* f. sp. *tritici*. Plant Dis 91:1096–1099. 10.1094/PDIS-91-9-1096 30780647

[CR20] Jin Y, Szabo LJ, Pretorius ZA et al (2008) Detection of virulence to resistance gene *Sr24* within race TTKS of *Puccinia graminis* f. sp. *tritici*. Plant Dis 92:923–926. 10.1094/PDIS-92-6-0923 30769714

[CR21] Karelov A, Kozub N, Sozinova O et al (2022) Wheat genes associated with different types of resistance against stem rust (*Puccinia graminis* Pers.). Pathogens 11:1157. 10.3390/pathogens11101157 PMC9608978 36297214

[CR22] Kaur S, Gill HS, Breiland M et al (2023) Identification of leaf rust resistance loci in a geographically diverse panel of wheat using genome-wide association analysis. Front Plant Sci 14:1090163. 10.3389/fpls.2023.1090163 PMC9929074 36818858

[CR23] Kerber ER, Dyck PL (1973) Inheritance of stem rust resistance transferred from diploid wheat (*Triticum monococcum*) to tetraploid and hexaploid wheat and chromosome location of the gene involved. Can J Genet Cytol 15:397–409. 10.1139/g73-050

[CR24] Knott DR, Anderson RG (1956) The inheritance of rust resistance.: I. The inheritance of stem rust resistance in ten varieties of common wheat. Can J Agric Sci 36:174–195. 10.4141/agsci-1956-0022

[CR25] Kolmer JA, Jin Y, Long DL (2007) Wheat leaf and stem rust in the United States. Aust J Agric Res 58:631–638. 10.1071/AR07057

[CR26] Liu X, Huang M, Fan B et al (2016) Iterative usage of fixed and random effect models for powerful and efficient genome-wide association studies. PLoS Genet 12:e1005767. 10.1371/journal.pgen.1005767 PMC4734661 26828793

[CR27] McIntosh R (1972) Cytogenetical studies in wheat VI. Chromosome location and linkage studies involving *Sr13* and *Sr8* for reaction to *Puccinia graminis* F. Sp. *tritici*. Aust J Biol Sci 25:765–774. 10.1071/BI9720765

[CR28] McIntosh R, Dyck P, Green G (1977) Inheritance of leaf rust and stem rust resistances in wheat cultivars Agent and Agatha. Aust J Agric Res 28:37–45. 10.1071/AR9770037

[CR29] McIntosh RA, Wellings CR, Park RF (1995) Wheat rusts: an atlas of resistance genes. CSIRO; Kluwer Academic Publishers, East Melbourne, Australia, Dordrecht

[CR30] Mettin D, Bulthner WD, Schlegel G (1973) Additional evidence on spontaneous 1B/1R wheat-rye substitutions and translocated. In: Proceedings of international wheat genetics symposium. Missouri Agricultural Experiment Station, Columbia, MO, USA, pp 179

[CR31] Money D, Gardner K, Migicovsky Z et al (2015) LinkImpute: fast and accurate genotype imputation for nonmodel organisms. G3 Genes Genomes Genet 5:2383–2390. 10.1534/G3.115.021667/-/DC1 PMC4632058 26377960

[CR32] Negash T, Edae EA, Tilahun L et al (2022) Genome‐wide association mapping for field and seedling resistance to the emerging *Puccinia graminis* f. sp. *tritici* race TTRTF in wheat. Plant Genome 15:e20274. 10.1002/tpg2.20274 PMC12807250 36263894

[CR33] Newcomb M, Olivera PD, Rouse MN et al (2016) Kenyan isolates of *Puccinia graminis* f. sp. *tritici* from 2008 to 2014: virulence to *SrTmp* in the Ug99 race group and implications for breeding programs. Phytopathology 106:729–736. 10.1094/PHYTO-12-15-0337-R 27019064

[CR34] Nirmala J, Chao S, Olivera P et al (2016) Markers linked to wheat stem rust resistance gene *Sr11* effective to *Puccinia graminis* f. sp. *tritici* race TKTTF. Phytopathology 106:1352–1358. 10.1094/PHYTO-04-16-0165-R 27359266

[CR35] Olivera P, Newcomb M, Szabo LJ et al (2015) Phenotypic and genotypic characterization of race TKTTF of *Puccinia graminis* f. sp. *tritici* that caused a wheat stem rust epidemic in Southern Ethiopia in 2013–14. Phytopathology 105:917–928. 10.1094/PHYTO-11-14-0302-FI 25775107

[CR36] Olivera PD, Hailu E, Wanyera R et al (2022a) TTKTT+: A new variant of the Ug99 race group detected in East Africa. In: Phytopathology®. P S3.1-S3.199

[CR37] Olivera PD, Jin Y, Rouse M et al (2012) Races of *Puccinia graminis* f. sp. *tritici* with combined virulence to *Sr13* and *Sr9e* in a field stem rust screening nursery in Ethiopia. Plant Dis 96:623–628. 10.1094/PDIS-09-11-0793 30727519

[CR38] Olivera PD, Newcomb M, Flath K et al (2017) Characterization of *Puccinia graminis* f. sp. *tritici* isolates derived from an unusual wheat stem rust outbreak in Germany in 2013. Plant Pathol 66:1258–1266. 10.1111/ppa.12674

[CR39] Olivera PD, Sikharulidze Z, Dumbadze R et al (2019) Presence of a sexual population of *Puccinia graminis* f. sp. *tritici* in Georgia provides a hotspot for genotypic and phenotypic diversity. Phytopathology 109:2152–2160. 10.1094/PHYTO-06-19-0186-R 31339468

[CR40] Olivera PD, Szabo LJ, Kokhmetova A et al (2022b) *Puccinia graminis* f. sp. *tritici* population causing recent wheat stem rust epidemics in Kazakhstan is highly diverse and includes novel virulence pathotypes. Phytopathology 112:2403–2415. 10.1094/PHYTO-08-21-0320-R 35671480

[CR41] Olson EL, Brown-Guedira G, Marshall D et al (2010) Development of wheat lines having a small introgressed segment carrying stem rust resistance gene *Sr22*. Crop Sci 50:1823–1830. 10.2135/cropsci2009.11.0652

[CR42] Patpour M, Baidya S, Basnet R et al (2024) First report of Ug99 wheat stem rust caused by *Puccinia graminis* f. sp. *tritici* in South Asia. Plant Dis 108:2570. 10.1094/PDIS-03-24-0644-PDN

[CR43] Peterson PD (ed) (2001) Stem rust of wheat: from ancient enemy to modern foe. APS Press, St. Paul, Minn

[CR44] Peterson RF, Campbell AB, Hannah AE (1948) A diagrammatic scale for estimating rust intensity on leaves and stems of cereals. Can J Res 26c:496–500. 10.1139/cjr48c-033

[CR45] Pretorius ZA, Singh RP, Wagoire WW, Payne TS (2000) Detection of virulence to wheat stem rust resistance gene *Sr31* in *Puccinia graminis* f. sp. *tritici* in Uganda. Plant Dis 84:203. 10.1094/PDIS.2000.84.2.203B 30841334

[CR46] Pujol V, Forrest KL, Zhang P et al (2015) Identification of a stem rust resistance locus effective against Ug99 on wheat chromosome 7AL using a RAD-Seq approach. Theor Appl Genet 128:1397–1405. 10.1007/s00122-015-2514-0 25877521

[CR47] R Core Team (2025) R: A language and environment for statistical computing. R Foundation for Statistical Computing, Vienna, Austria

[CR48] Rasheed A, Wen W, Gao F et al (2016) Development and validation of KASP assays for genes underpinning key economic traits in bread wheat. Theor Appl Genet 129:1843–1860. 10.1007/s00122-016-2743-x 27306516

[CR49] Rauf Y, Bajgain P, Rouse MN et al (2022) Molecular characterization of genomic regions for adult plant resistance to stem rust in a spring wheat mapping population. Plant Dis 106:439–450. 10.1094/PDIS-03-21-0672-RE 34353123

[CR50] Roelfs AP (1985) Wheat and rey stem rust. In: Roelfs AP, Bushnell WR (eds) The cereal rusts (Vol II) Diseases, distribution, epidemiology, and control. Academic Press, Orlando, pp 3–37

[CR51] Roelfs AP, Singh RP, Saari EE (1992) Rust diseases of wheat: concepts and methods of disease management. CIMMYT, Mexico, D.F

[CR52] Rouse MN, Nirmala J, Jin Y et al (2014) Characterization of *Sr9h*, a wheat stem rust resistance allele effective to Ug99. Theor Appl Genet 127:1681–1688. 10.1007/s00122-014-2330-y 24913360

[CR53] Rouse MN, Wanyera R, Njau P, Jin Y (2011) Sources of resistance to stem rust race Ug99 in spring wheat germplasm. Plant Dis 95:762–766. 10.1094/PDIS-12-10-0940 30731910

[CR54] Sallam AH, Conley E, Prakapenka D et al (2020) Improving prediction accuracy using multi-allelic haplotype prediction and training population optimization in wheat. G3 Genes Genomes Genet 10:2265–2273. 10.1534/G3.120.401165 PMC7341132 32371453

[CR55] Shahin A, Mazrou YSA, Omara RI et al (2022) Geographical correlation and genetic diversity of newly emerged races within the Ug99 lineage of stem rust pathogen, *Puccinia graminis* f. sp. *tritici*, in different wheat-producing areas. J Fungi 8:1041. 10.3390/jof8101041 PMC9605546 36294606

[CR56] Singh RP, Hodson DP, Huerta-Espino J et al (2011) The emergence of Ug99 races of the stem rust fungus is a threat to world wheat production. Annu Rev Phytopathol 49:465–481. 10.1146/annurev-phyto-072910-095423 21568701

[CR57] Singh RP, Hodson DP, Huerta-Espino J et al (2008) Will stem rust destroy the world’s wheat crop? Adv Agro 98:271–309

[CR58] Singh RP, Huerta-Espino J, Roelfs AP (2002) The wheat rusts. Growth 2(25):35

[CR59] Singh RP, Singh PK, Rutkoski J et al (2016) Disease impact on wheat yield potential and prospects of genetic control. Annu Rev Phytopathol 54:303–322. 10.1146/annurev-phyto-080615-095835 27296137

[CR60] Stakman EC (1923) Barberry eradication prevents black rust in Western Europe. United States Department of Agriculture

[CR61] Stakman EC, Stewart DM, Loegering WQ (1962) Identification of physiologic races of *Puccinia graminis* var. *tritici*. United States Department of Agriculture. E617. Washington, DC

[CR62] Stuthman DD, Leonard KJ, Miller‐Garvin J (2007) Breeding crops for durable resistance to disease. Advances in agronomy. Elsevier, pp 319–367

[CR63] Tong J, Zhao C, Liu D et al (2024) Genome-wide atlas of rust resistance loci in wheat. Theor Appl Genet 137:179. 10.1007/s00122-024-04689-8 PMC11233289 38980436

[CR64] Wang J, Zhang Z (2021) GAPIT version 3: boosting power and accuracy for genomic association and prediction. Genom Proteom Bioinform. 10.1016/j.gpb.2021.08.005 PMC9121400 34492338

[CR65] Wang S, Wong D, Forrest K et al (2014) Characterization of polyploid wheat genomic diversity using a high-density 90 000 single nucleotide polymorphism array. Plant Biotechnol J 12:787–796. 10.1111/pbi.12183 PMC4265271 24646323

[CR66] Wanyera R, Kinyua MG, Jin Y, Singh RP (2006) The spread of stem rust caused by *Puccinia graminis* f. sp. *tritici*, with virulence on *Sr31* in wheat in Eastern Africa. Plant Dis 90:113–113. 10.1094/PD-90-0113A 30786499

[CR67] Watson IA, Luig NH (1966) *Sr15* — a new gene for use in the classification of *Puccinia graminis* var. *tritici*. Euphytica 15:239–247. 10.1007/BF00022329

[CR68] Wickham H (2016) Ggplot2: elegant graphics for data analysis. Springer-Verlag, New York

[CR69] Yu J, Pressoir G, Briggs WH et al (2006) A unified mixed-model method for association mapping that accounts for multiple levels of relatedness. Nat Genet 38:203–208. 10.1038/ng1702 16380716

